# A patient-mediated implementation strategy to improve nutrition care delivery for esophageal and gastric cancer: a study protocol for a pilot randomized controlled trial

**DOI:** 10.1186/s41043-025-01208-3

**Published:** 2025-12-28

**Authors:** Kea Turner, Ashwin Somasundaram, Brent J. Small, Jeanine Milano, Christina Santiago, Olivia Sprow, Emma Hume, Nazanin Khajoueinejad, Nekesha McKinnie, Allan Lima Pereira, Andrew Sinnamon, Jennifer B. Permuth, Amir Alishahi Tabriz, Jose M. Pimiento

**Affiliations:** 1 University of North Carolina at Chapel Hill School of Nursing, 211 Manning Drive, Chapel Hill, NC 27599 USA; 2 University of North Carolina at Chapel Hill Lineberger Comprehensive Cancer Center, 450 West Drive, Chapel Hill, NC 27599 USA; 3 Department of Medicine, University of North Carolina at Chapel Hill School of Medicine, NC 321 South Columbia Street, Chapel Hill, 27514 USA; 4 Department of Nutrition Therapy, Moffitt Cancer Center, 12902 USF Magnolia Drive, Tampa, FL 33612 USA; 5 Department of Gastrointestinal Oncology, Moffitt Cancer Center, 12902 USF Magnolia Drive, Tampa, FL 33612 USA; 6 Department of Cancer Epidemiology, Moffitt Cancer Center, 12902 USF Magnolia Drive, Tampa, FL 33612 USA; 7 Department of Health Outcomes and Behavior, Moffitt Cancer Center, 12902 USF Magnolia Drive, Tampa, FL 33612 USA

**Keywords:** Oncology, Nutrition, Gastrointestinal oncology, Patient-mediated implementation, Patient activation

## Abstract

**Background:**

Nutrition counseling before gastrointestinal surgery can improve patients’ quality of life, treatment tolerance, and surgical recovery. Nutrition counseling is especially important for individuals diagnosed with esophageal and gastric cancer, who are at high risk of malnutrition. Despite the increased risk for malnutrition, less than half of individuals diagnosed with esophageal and gastric cancer receive nutrition counseling before surgery. To address this practice gap, the Support through Remote Observation and Nutrition Guidance (STRONG) implementation strategy was developed.

**Methods:**

STRONG includes three theory-informed strategies: an electronic health record order set for a standardized protocol that specifies the timing and amount of nutrition counseling that should be received, collection and visualization of patient-reported nutrition information in a web-based dashboard, and a question prompt list for the patient-dietitian encounter. Patients (*N* = 80) will be 1:1 randomized to STRONG or implementation as usual in this pilot trial. The trial will be pragmatic in design and STRONG will be implemented with existing clinic teams and workflows. The study’s primary aim will be to assess feasibility and acceptability against pre-planned benchmarks and secondary aims include collecting preliminary data on effectiveness and implementation outcomes that will support a future definitive hybrid implementation-effectiveness trial.

**Discussion:**

Pilot studies play a critical role in implementation research by ensuring that strategies and proposed study methods are feasible prior to conducting a definitive trial. Positive findings from this line of research could support definitive testing of a patient-mediated implementation strategy to improve nutrition care access and delivery for cancer patients at high risk of malnutrition.

**Trial registration:**

ClinicalTrials.gov NCT06497569. Registered on July 11, 2024, prior to participant enrollment.

## Contributions to the Literature

Patient-mediated implementation strategies aim to improve the delivery of healthcare through patient engagement and can affect patient outcomes and healthcare delivery

There has been limited study of patient-mediated implementation strategies in implementation science

Existing studies have focused on the impact of patient-mediated strategies on patient outcomes and not examined implementation or service delivery outcomes

This protocol outlines a pilot study of a patient-mediated implementation strategy to improve patient outcomes and nutrition care delivery in oncology

This line of research will lay the groundwork for a future definitive trial that evaluates the strategy’s effect on patient outcomes and nutrition care delivery

## Background

Studies estimate that most esophageal and gastric cancer patients are diagnosed with malnutrition during cancer treatment [1–6]. The high prevalence of malnutrition is caused by the cancer’s location in the digestive tract and receipt of cancer treatments that affect nutrition [7–10]. Esophageal and gastric cancer patients may experience nutrition-impact symptoms, such as swallowing difficulty, that affect the ability and desire to eat [7, 11, 12]. Patients can also experience problems with digestion, motility, and nutrient absorption [13–15]. Malnutrition has a negative impact on patients’ quality of life, treatment tolerance, and survival and increases caregiver distress and burden [9, 16–24]. At the system level, malnutrition can increase hospital length of stay, readmission rates, and healthcare costs [25–27]. Clinical guidelines recommend that all cancer patients at risk for malnutrition receive nutrition counseling from a registered dietitian [28–32], an evidence-based intervention for improving nutritional status [33, 34]. In practice, access and delivery of nutrition counseling for patients with malnutrition varies across cancer care settings [35–38]. 

Despite the importance of nutrition counseling, studies suggest that less than half of esophageal and gastric cancer patients meet with a dietitian during cancer treatment [39, 40]. Nutrition counseling is particularly important for patients who are receiving surgical treatment. Patients receiving surgical treatment are likely to receive neoadjuvant treatment to prepare for surgery, such as chemotherapy and radiotherapy, which can worsen nutritional status [41–43]. Nutritional status is a key predictor of post-surgical complications and recovery [44–47]. Therefore, intervening on nutritional status prior to surgery is critical for improving patient outcomes. To improve nutrition care delivery prior to surgery, several barriers need to be overcome. First, many cancer care systems lack standardized protocols that specify when dietitian referrals should be made or how much counseling is needed, resulting in late referrals and inadequate follow-up [36, 48–50]. Second, dietitians may lack sufficient patient-reported information, such as nutrition-impact symptoms, that may be important for monitoring malnutrition and tailoring nutrition counseling to the individual patient [48, 51, 52]. Third, patients may not understand the role of the dietitian in cancer care or the potential value of nutrition counseling. Given the many competing priorities cancer patients face, especially early on during cancer treatment, patients may be reluctant to meet with a dietitian when referred [53, 54]. Finally, patients may not have the skills or resources necessary to engage in and adhere to recommendations from nutrition counseling (e.g., tracking dietary goals, food security) [55–60]. To ensure consistent access and delivery of nutrition counseling, implementation strategies are needed to standardize early and ongoing nutrition counseling, integrate patient-reported nutrition information into the clinical workflow, and provide patient-mediated tools to empower patients to actively participate in their nutrition care.

To address this gap, our study team developed Support through Remote Observation and Nutrition Guidance (STRONG), a novel patient-mediated strategy to improve access and delivery of nutrition counseling. The target population is individuals diagnosed with esophageal and gastric cancer who are preparing for surgery. We chose this population given the high burden of malnutrition [1–6] and the potential to deliver nutrition counseling early when impact on patient outcomes may be maximized [44–47]. The long-term goal of this line of research is to improve the quality of nutrition care that cancer patients receive and to reduce the adverse outcomes for patients, caregivers, and cancer care systems that are associated with malnutrition. This manuscript will present the methodology of a pilot randomized controlled trial that compares the STRONG program with implementation as usual. The trial is designed to be pragmatic with broad inclusion criteria and use of existing clinical staff and pathways [61]. The primary aim of the study is to assess the feasibility and acceptability of the STRONG program among esophageal and gastric cancer patients, cancer caregivers, and healthcare providers. A secondary aim is to collect preliminary data on implementation outcomes guided by the RE-AIM (reach, effectiveness, adoption, implementation and maintenance) framework [62] and effectiveness outcomes (patient and service delivery) [63] that may be improved by STRONG. Findings from this pilot study will be used to refine the STRONG implementation strategy and support a future definitive hybrid implementation trial. Further, there has been limited study of patient-mediated strategies within implementation science. To date, most studies have evaluated the impact of patient-mediated strategies on patient outcomes and have not measured the impact on implementation outcomes or service delivery [64–67]. Research from the proposed study will contribute to the literature by advancing our understanding of how patient-mediated implementation strategies affect the implementation and delivery of nutrition care in oncology.

## Methods

### Study setting

The study will be conducted at Moffitt Cancer Center (Moffitt) and the University of North Carolina at Chapel Hill (UNC), two Cancer Centers that serve different patient populations. Moffitt is a standalone, non-profit National Cancer Institute (NCI)designated Comprehensive Cancer Center that serves a primarily urban patient population (93%) in a region of Florida where 21% of the population is Latino/Hispanic. UNC is a public academic medical center that includes a matrix NCI-designated Comprehensive Cancer Center and affiliated community oncology clinics. UNC serves a patient population that is ~ 33% rural and 20% Non-Hispanic Black/African American.

### Study design

The study will use a parallel pilot randomized controlled trial design and collect preliminary data on implementation and effectiveness outcomes. Patients will be 1:1 randomized to the 12-week STRONG program versus usual care and complete study assessments at baseline, 1 month, 3 months (end of intervention), and 6 months. The protocol is reported in accordance with the SPIRIT 2025 guidelines and Table 1 provides a study timeline [68]. Data will also be collected from healthcare providers (e.g., dietitians, physicians, nurses) and caregivers who support implementation. The trial is designed to be pragmatic and has broad inclusion criteria and implementation is integrated within existing care teams and workflows [61].

**Table 1:**
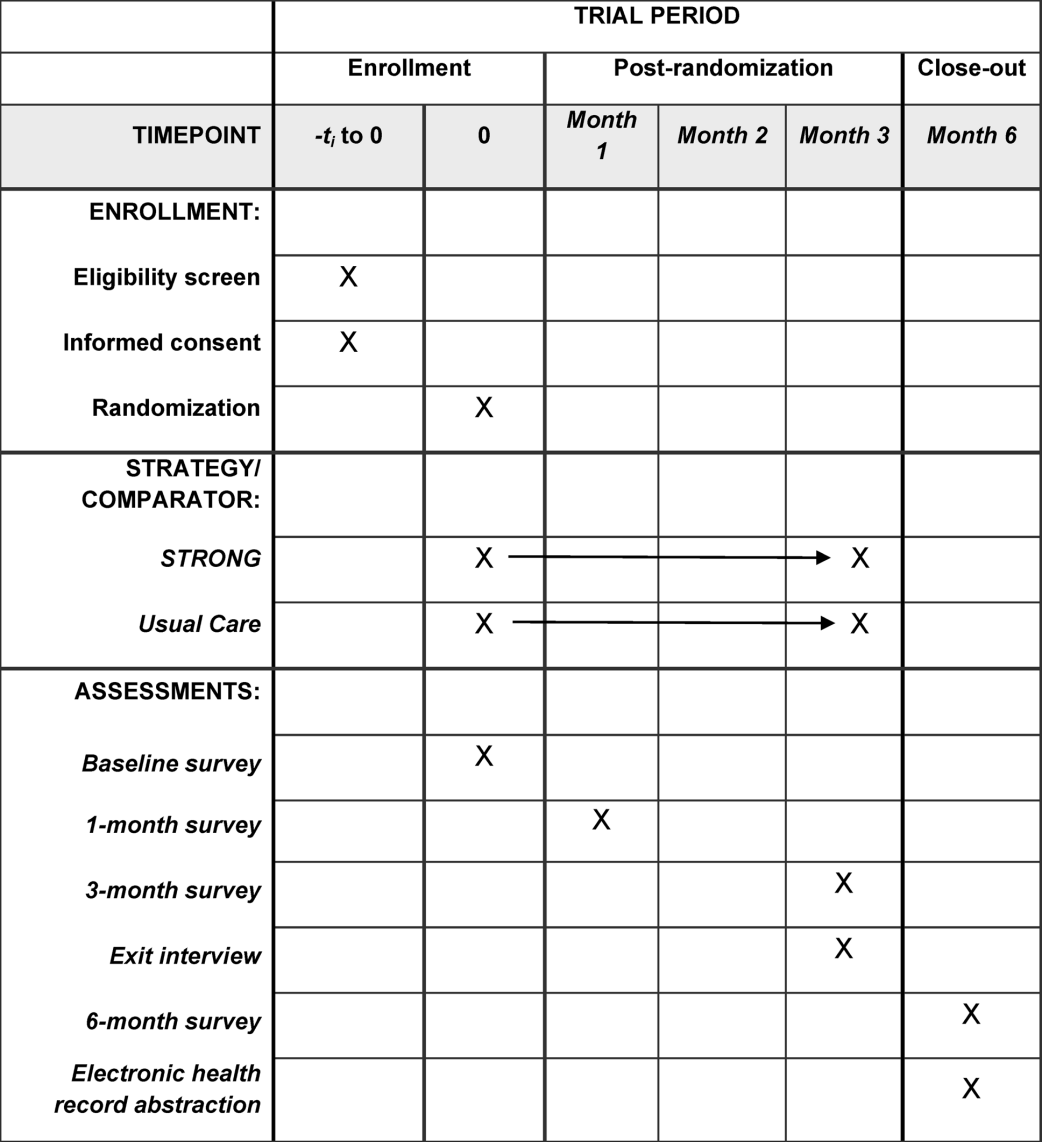
Study timeline: Schedule of enrollment, interventions, and assessments

### Using the theoretical domains framework to develop a patient-mediated implementation strategy

The Theoretical Domains Framework (TDF) identifies the psychological, social, and environmental determinants that affect behavior change and implementation of evidence-based care [69–71]. The study team conducted a systematic review and qualitative research with patients, caregivers, and clinicians to identify key dietitian and patient behaviors to be targeted and potential determinants of behavior change, which were categorized based on the TDF. STRONG targets four primary barriers to nutrition counseling access and delivery including (1) lack of standardized protocols for dietitian referral and follow-up; (2) lack of patient-reported nutrition information to assist dietitians’ with monitoring malnutrition and tailoring nutrition counseling; (3) lack of patient awareness about the role of the dietitian in cancer care and the perceived value of nutrition care; and (4) lack of patient skills and resources necessary for engaging in and adhering to nutrition counseling recommendations. These barriers were mapped to the Theoretical Domains Framework (TDF) [69–71] and linked with the Behavior Change Technique Taxonomy [72], which informed the selection of implementation strategies.

**Fig. 1:**
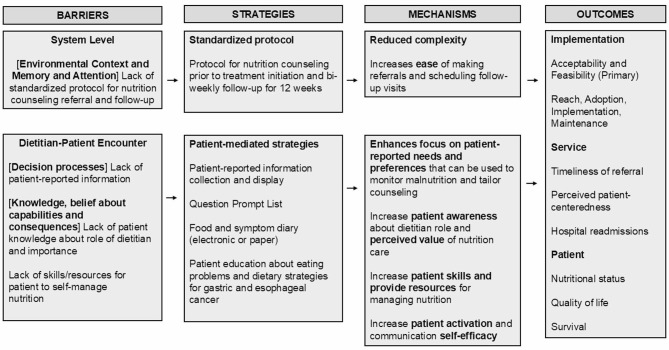
STRONG implementation logic model based on the theoritical domains framework

The implementation logic model [73] for STRONG is presented in Fig. 1. The intervention includes an electronic health record (EHR) order for a standardized nutrition protocol that includes early nutrition counseling (first referral prior to treatment initiation) and ongoing follow up (bi-weekly visits for the first 12 weeks of treatment). The order set is designed to reduce complexity for the clinician by creating an automatic schedule of follow-up visits rather than ad-hoc scheduling of follow-up appointments that vary for each patient. Prior studies suggest that standardized protocols, particularly when available in the EHR, can increase the timeliness of referrals for evidence-based care [74–78]. 

Referrals are more effective when paired with patient-mediated strategies that prepare the patient for engaging in the clinical encounter. Previous research has demonstrated that education about the purpose of a clinical visit and its potential value may increase uptake of nutrition and supportive care among cancer patients [50, 79]. The study team developed a question prompt list and a ‘what to expect from your dietitian visit’ handout to prepare patients for the dietitian visit. In other settings, question prompt lists have been effective for improving patient activation, communication self-efficacy and participation in clinical encounters (e.g., asking relevant questions) [80–84]. Patients will also receive a self-guided education booklet about eating problems and coping strategies specific to gastric and esophageal cancer to reinforce content that is provided as a part of nutrition counseling.

Research suggests that dietitians may need more patient-reported information to effectively monitor malnutrition and tailor nutrition counseling [51, 59, 85–88]. To address this, patient-reported information is collected from the patient including nutritional status (via the PG-SGA short-form [89, 90]), food intake and nutrition symptoms, and patient needs and preferences (e.g., food insecurity via Hunger Vital Sign [91]). Information collected is shared with a dietitian via a web-based dashboard and patients reporting food insecurity are provided with the option for referral to a nurse navigator or social worker. This information is collected to provide dietitians with data they may not otherwise have access to and help focus the nutrition counseling visits on patients’ needs and preferences (e.g., reviewing a food log from the prior week to identify opportunities for eating more nutrient dense foods when calorie goals are not being met). Prior studies have demonstrated that patient-reported information at the clinic encounter can improve the patient-centeredness of care and clinician’s ability to deliver timely and individualized feedback [92–95]. 

The long-term goal of the program is to improve access and consistency in the delivery of nutrition counseling, leading to improvements in service delivery (e.g., timeliness and patient-centeredness of nutrition care, hospital readmissions) and patient outcomes (e.g., nutritional status, quality of life, treatment tolerance, and survival) (Fig. 1).

### Trial approval, registration, and funding

The pilot trial is funded by the Department of Defense (DoD). The study protocol was approved by the Moffitt Institutional Review Board (IRB) of Record, Advarra, and a reliance agreement was established with the UNC IRB. The trial was registered on ClinicalTrials.gov (NCT06497569) prior to participant enrollment.

### Participant eligibility

Patients who meet the following criteria will be eligible: 1) ≥ 18 years of age; 2) diagnosis of gastric, esophageal, or gastroesophageal junction cancer; 3) planning to start systemic therapy and/or radiotherapy at the time of study enrollment; 4) have a treatment plan that includes surgery or definitive follow up at Moffitt or UNC; and 5) ability to provide informed consent in Spanish or English. Patients will be excluded if they have a hospice referral or plan for hospice enrollment at the time of study enrollment.

### Participant recruitment and consent

Study staff at both sites will screen the EHR for the minimum information necessary to identify potentially eligible patients with upcoming appointments and clinic staff will be encouraged to refer eligible patients to study staff. The study team will manage a Zoom channel that includes the dietitian and nursing team for communication and manage a study related inbox. At UNC, the Carolina Data Warehouse will develop a weekly automated screening list of potentially eligible patients from the UNC Health System (e.g., Lineberger Comprehensive Cancer Center and UNC affiliated community oncology sites). The study team will develop study flyers and a short video that describes the key components of the study to support recruitment.

Research staff who have completed human subjects and Health Insurance Portability and Accountability Act (HIPAA) training will perform screening and consent. Research staff will approach potentially eligible patients in clinic or contact them by phone or patient portal using an IRB-approved message to gauge interest. Study staff will introduce the study, assess interest, review key elements of informed consent, and obtain written informed consent from interested patients. A HIPAA authorization will be obtained from individuals who consent to participate in the study. A waiver of HIPAA authorization will be obtained for EHR screening and to retain a limited set of information on patients who decline to participate (e.g., sex, race/ethnicity, age, language preference, reason for decline) so that individuals who consent can be compared with individuals who declined to participate.

### Randomization

Patients (*N* = 80) will be 1:1 randomized to the STRONG program versus usual care. The study statistician will develop a randomization table using the randomization function in Stata (StataCorp. 2025. Stata Statistical Software: Release 19. College Station, TX: StataCorp LC.) that will be uploaded to REDCap^®^ (Research Electronic Data Capture). Randomization will be stratified based on cancer type and treatment modality (e.g., single versus multimodal treatment) to ensure groups are balanced on these factors, which may affect malnutrition risk. Block randomization (in variable block sizes of 4 and 6) will be used to maintain balance across groups and concealment. Outcome assessors and the study statistician will be blinded to group allocation.

### Intervention condition

Prior to treatment initiation, patients randomized to the intervention condition will meet with a study coordinator to receive and review the education brochure on common eating problems related to gastric and esophageal cancer and potential coping strategies, the question prompt list, and the handout on what to expect during a dietitian visit. Patients will be encouraged to bring a caregiver when available. Patients will also be asked about their preferences for recording nutrition-impact symptoms and food intake and be provided with an electronic option (food logging through Fitbit Inspire 3.0 and texting messaging tool) or a paper option (paper food diary). The research coordinator will review how to use either the electronic or paper option and use the teach-back method to ensure the participant understands how to use the tools. For patients using Fitbit to electronically record food intake, the study team will create a Fitbit account using a pseudonym (e.g., participant 1) and a study provided email address so that no identifying information is entered into the Fitbit app. The Fitbit app will be downloaded to the patients’ smartphone or a study-loaned device (e.g., cellularly enabled tablet) for patients who do not have a smart device or do not want to use their smart device for the study (e.g., limited data plan). The study coordinator will contact the clinic team, who will place the EHR order for the standardized nutrition counseling protocol. The protocol includes an initial dietitian visit prior to treatment initiation and bi-weekly follow-up for 12 weeks.

The first nutrition counseling appointment will be an hour in length after the patient has been discharged. The nutrition counseling will be delivered using the HIPAA-compliant version of Zoom videoconference; however, participants will have the option to schedule a phone visit or in-person visit if preferred. The content of the visit is based on nutrition guidelines in oncology and feedback gathered from clinicians, patients, and caregivers from qualitative research (Table 2) [28, 96–98]. Calorie and protein goals will be based on the European Society for Clinical Nutrition and Metabolism (ESPEN) guideline for clinical nutrition in cancer [28] and may be modified based on dietitian judgement (e.g., patient activity level, malnutrition severity). After the initial visit, the patient will meet with the dietitian on a bi-weekly basis. Follow-up visits will be approximately 30 min. Patients will use the app (or paper log) between visits to log food intake and nutrition symptoms at least 3x a week (non-consecutive days) and complete a malnutrition screening (PG-SGA short form [89, 90]) monthly. The intervention will last for the first 12 weeks (3 months) of treatment (when nutrition impact symptoms often increase in severity) [99, 100]. Guidance from NCI and prior studies of dietary assessment suggest that 3 days of non-consecutive food records is sufficient for accurately estimating food intake and reduces participant burden (compared with daily food records) [101, 102]. Therefore, we will encourage patients to log food intake and nutrition symptoms 3 days a week rather than daily. Dietitians will be given access to a dashboard that displays patient-reported information (e.g., malnutrition screening, food intake, nutrition impact symptoms). Visits will be documented in the EHR, which contains a template that covers key visit topics (e.g., nutrition history, food preferences).

### Usual care condition

Patients randomized to the usual care condition will receive usual implementation for nutrition counseling, which means that a member of the clinic team will refer patients to a dietitian when they deem it necessary. Patients in the usual care condition will receive the same educational brochure that the intervention group receives about eating problems that occur during cancer treatment targeted for gastric and esophageal cancer patients. Nutrition education is considered a part of standard of care; therefore, the educational booklet developed will also be provided to the control group.

### Retention strategies

To optimize retention, patients can choose to receive the intervention in a format they prefer (app or paper log). We will engage caregivers in implementation of patient-mediated strategies when available and provide training, technical assistance, and follow-up. Study staff will train patient participants, call all patients within 3–5 days of study enrollment to confirm understanding of what is being asked of them and see if they need any help. Study staff will help patient participants troubleshoot any technical challenges (e.g., REDCap survey not working) and refer patients to additional resources when needed (e.g., Zoom help line managed by the Cancer Center). Patients who opt in for study-related text messaging will receive reminders for upcoming dietitian appointments and study-related tasks (e.g., patient-reported outcome reporting). We will provide patients with summaries about their study progress (e.g., 75% of dietitian visits are completed).

### Measures

#### Study assessment schedule

Patients will receive a survey at baseline, 1 month, 3 months (end of intervention), and 6 months. Data will also be obtained from patient-reported information collection (app or paper log), and the EHR. Patients will have the option to participate in a one-time exit interview to provide feedback on the intervention (nutrition counseling) and the patient-mediated implementation strategies (e.g., patient-reported information collection, education, question prompt list). The study team will also collect data from caregivers and healthcare providers (e.g., dietitians, physicians, nurses) who assisted with study implementation through a one-time exit interview. Patient participants will receive $25 gift cards for each study assessment completed and a $50 gift card for interview participation. Study assessments and materials including surveys and interview guides have been translated and are available in Spanish and English.

### Primary outcomes

Primary outcomes for this pilot study are feasibility and acceptability. Feasibility will be defined based on (1) recruitment rate (proportion of eligible patients who consent); (2) study assessment completion; (3) intervention adherence and engagement (app or paper log use, participation in dietitian visits, use of question prompt list and handout); (4) retention rate (proportion of patients who are retained at the end of the intervention); and (5) reasons for declining to participate and study attrition. Acceptability will be measured based on the proportion of patients who report satisfaction with the overall intervention and individual intervention components. Outcomes will be measured through study screening logs and administrative data (e.g., completed assessments in REDCap). We will compare feasibility and acceptability outcomes observed in the proposed study against pre-planned benchmarks (Table 3), which were established based on prior studies in similar patient populations [103–108]. 

### Secondary implementation outcomes

The study will also collect preliminary data on implementation outcomes that may be associated with the STRONG program based on the RE-AIM framework [62] including reach (proportion of target population that was reached by the intervention), adoption (proportion of providers who referred patients to the study), implementation (fidelity, contamination, adaptation), and maintenance (impact of the intervention at six months). Fidelity will be measured to determine if key elements of the intervention were delivered (e.g., establishing individual calorie goals, discussing patients’ goals for improving nutrition) and how much of the intervention was delivered (e.g., length of dietitian visits, participant use of strategies recommended by the dietitian). We will document potential contamination (ask usual care patient participants about use of patient-mediated strategies), and unplanned intervention modifications using the FRAME documentation tool [109]. 

### Qualitative feedback

A subset of patient participants (*N* = 20; ~10 per site) will be asked to provide feedback on the quality of the intervention (e.g., nutrition counseling) and strategies (e.g., question prompt list, patient-reported information), and any barriers/facilitators to intervention uptake and/or strategy implementation. Caregivers and healthcare providers who supported implemented (*N* = 25; ~5 per group [dietitian, physician, nurse, caregiver]) will be asked to provide feedback on the implementation strategies and barriers and facilitators to implementation. The interviews will be led by a staff member trained in qualitative methods using a semi-structured interview guide based on the TDF [69–71]. The interviews will be approximately 30–45 min in length, recorded, and transcribed verbatim.

### Secondary effectiveness outcomes

The study will collect preliminary data on patient and service delivery outcomes that may be associated with the STRONG program. Nutritional status will be measured using the PG-SGA short-form and serve as the primary effectiveness outcome in a future definitive trial [89, 90]. A change in score of 4 points is considered clinically meaningful [89, 90]. We will examine changes in the proportion of patients who are categorized as malnourished via the PG-SGA based on cutoffs established in prior research [110]. We will assess other indicators of nutritional status including changes in weight [111] and skeletal muscle mass. Skeletal muscle mass will be estimated from routinely collected CT scans and categorized based on previously established cutoffs for sarcopenia [112]. We will use CT ‘slices’ from the midpoint of the third lumbar vertebrae for analyses. We will blind CT assessors to study arm. Quality of life will be measured using 1) the Functional Assessment of Cancer Therapy General scale [113]; 2) the Functional Assessment of Anorexia/Cachexia Therapy subscale [114]; and 3) the FACT–esophageal and gastric cancer subscale [115, 116]. Change in quality-of-life scores will be measured using established minimal clinically important differences (MCIDs) when available [117–119]. We will measure planned versus received systemic therapy and/or radiotherapy to define treatment delay (yes/no), dose reduction (yes/no), or treatment discontinuation (yes/no). To measure survival, we will obtain vital status from the cancer registry to estimate overall survival (time from random assignment to death from any cause) and progression-free survival (time from random assignment to disease progression or death from any cause). We will measure service delivery outcomes including the timeliness of dietitian referrals (e.g., median time from diagnosis to first referral) and patient perceptions about the patient-centeredness of nutrition care delivery using the Patient-Centered Communication in Cancer Care Short Form (6-item) [120]. 

### Mechanisms of change

We will measure two factors that may serve as mechanisms of change and help explain the potential impact of STRONG on improved implementation of nutrition counseling. First, we will measure patient activation (knowledge, skills, and confidence in managing health and healthcare) via the Patient Activation Measure (13-item) [121, 122]. Second, we will measure patient self-efficacy for communicating with dietitians via the Perceived Self-Efficacy Scale [123]. 

### Participant and treatment characteristics

Participant characteristics will be captured via the baseline survey and include factors that may affect engagement with the patient-mediated implementation strategy (e.g., sex, age, race/ethnicity, language preference, digital and health literacy). Clinical characteristics that may affect nutritional status (e.g., cancer stage, comorbidities) will be abstracted from the EHR. Treatment characteristics (e.g., single vs. multi-modal treatment, treatment type, dose planned versus dose received) will be abstracted from the EHR. Nutrition-related interventions and date of delivery (e.g., feeding tube) will be abstracted.

### Data analyses

#### Analytic approach

The primary purpose of this trial is to estimate feasibility outcomes against pre-planned benchmarks to prepare for a future fully powered trial. A secondary goal of this pilot is to explore the preliminary effects of the intervention on patient outcomes. We will use an intent-to-treat approach and analyze all randomized patientsto explore real-world effectiveness and conduct additional per-protocol analysis to explore effects in patients who adhered to the study protocol. We will examine potential sources of bias (e.g., selection bias, differential attrition) and the amount and pattern of missing data (e.g., by arm/time) and adjust the statistical approach as needed. Plans for each Aim are described below.

#### Aim 1 analyses

Feasibility and acceptability outcomes will be summarized using descriptive statistics and compared against pre-planned benchmarks. We will use thematic analyses to analyze participant exit interview transcripts [124]. We will use a hybrid approach [125] for the thematic analyses by developing deductive codes based on the interview guide [126] and inductive codes that emerge from the data [127]. Two individuals will code all transcripts and come to a consensus regarding coding. We will use peer debriefs to discuss coding and interpretation, write memos to record reflections, and triangulate qualitative data with the quantitative data [128, 129]. Data gathered will be used to refine the implementation strategy in future studies.

#### Aim 2 analyses

Preliminary outcomes will be measured at three time points (baseline, 3-, and 6-months) for two groups (intervention and control). We will use linear mixed-effects models to estimate changes in PG-SGA scores between groups, as they account for dependence of longitudinal assessments and allow for patients with incomplete data to be included in analyses. The model will include fixed effects for time, group, and group X time. A group X time interaction will provide evidence for differential change by treatment groups. Randomization strata and any group differences not balanced through randomization procedures will be included as covariates in the model. Recruitment site (UNC, Moffitt) will be included as a random effect in the analyses. Mean differences will be compared against the threshold for a clinically significant difference in PG-SGA score (4-point change). A similar approach will be applied for other discrete or continuously measured outcomes using MCIDs when available. We will use generalized estimating equations (GEEs) to estimate whether the change in the proportion of patients with severe malnutrition (yes/no) over time differs between the intervention and control group. A similar approach will be applied for other binary outcomes. For time-to-event outcomes (i.e., survival), we will use a stratified Cox proportional hazards model (stratified by randomization strata) and adjusted for any characteristics not balanced across groups. The primary parameter of interest is the Cox Hazards Ratio (HR). We will test model assumptions (e.g., Schoenfield residuals) and adjust the statistical approach as needed (e.g., use of time-varying HR). We will model Kaplan-Meier curves by group assignment from baseline to 6-months.

#### Sample size considerations

The goal of this pilot randomized controlled trial is to assess feasibility of study procedures and feasibility of the implementation strategy prior to a definitive hybrid implementation-effectiveness trial. We will recruit 80 patients, assuming 15% will be lost due to attrition based on our prior studies, leaving a sample size of 68 (34 patients per arm). Guidance for conducting pilot randomized trials suggests 25–35 participants per arm is sufficient to estimate feasibility outcomes, such as recruitment and retention, and to provide preliminary estimates of intervention effect and variation [130–132]. Consistent with NIH guidance, this pilot trial is not powered for definitive hypothesis testing; the sample size selection is based on the goal of estimating feasibility outcomes and providing preliminary intervention effect estimates [133]. Nonetheless, with 68 patients, the study is powered to estimate mean change in PG-SGA score with reasonable precision (CI ± 2.4–2.8 points) depending on the standard deviation assumption (6–7 based on prior studies) [107] and an assumption of *r*=-.55 based on prior studies [134]. 

#### Data monitoring, analysis, and security

The PI and study staff will meet weekly to review study feasibility and acceptability (e.g., recruitment) and adherence to study procedures and potential protocol deviations. The study team will review other key quality metrics with the entire study team (e.g., participant satisfaction) on a quarterly basis. If any problems are observed (e.g., feasibility benchmark not obtained), the study team will review procedures and consider modifying the protocol if necessary. The proposed study is minimal risk and will not require external safety monitoring. Nonetheless, the study team will use the following procedures to monitor risk: (1) prompting patients to report any problems related to the study protocol (e.g., burden); (2) maintaining an adverse event log; and (3) following institutional and funder policies for prompt reporting of serious or unanticipated adverse events and pausing the study protocol if necessary. The study team will follow institutional data security procedures (e.g., minimizing use of protected health information) to protect participant data.

#### Design considerations

We chose to include all patients with a diagnosis of gastric or esophageal cancer given the high prevalence of malnutrition and nutrition-impact symptoms (e.g., swallowing difficulty) in this patient population, as opposed to screening for malnutrition and only including individuals who screen positive for malnutrition (e.g., a disease-based approach versus a triggered approach) [1–6]. Historically, a screen and referral approach has been recommended for all oncology patients but may not be fitting for patient populations where malnutrition is nearly universal (not something that varies across patients).

### Dissemination and future research

Study findings will be disseminated through required updates for ClinicalTrials.gov and a plain language summary of study findings that will be shared with patient participants, clinic team members, and other key stakeholders. Study findings will also be shared through conference abstracts and peer reviewed publications. Findings from the study will support a definitive hybrid-implementation trial. At the conclusion of the study, de-identified data will be deposited in the UNC Dataverse repository to facilitate data sharing.

### Patient, caregiver, and clinician involvement

The STRONG intervention was developed based on extensive data gathering and iterative testing with patients, caregivers, and clinicians through qualitative research and user-centered design, findings that will be reported elsewhere.

## Discussion

Pilot studies play a critical role in implementation research by ensuring that strategies and proposed study methods are feasible prior to conducting a larger and more costly definitive trial [135]. This pilot study will assess the feasibility of a patient-mediated implementation strategy to improve nutrition care delivery for esophageal and gastric cancer patients, who have a high burden of malnutrition [1–6]. The study is timed to deliver support to patients during neoadjuvant therapy prior to surgery, when nutrition care plays a pivotal role in long-term outcomes [44–47]. In the future, we aim to expand the program to individuals diagnosed with metastatic gastric and esophageal cancer who are receiving palliative chemotherapy, who may also greatly benefit from nutrition counseling. There has been limited research on implementation strategies designed to overcome barriers to delivering nutrition care during cancer treatment [136]. Additionally, there has been limited research on patient-mediated implementation strategies as an approach to increase evidence-based care delivery [66]. Positive findings from this line of research could expand the reach of evidence-based nutrition care during cancer treatment and improve patients’ nutritional status, quality of life, treatment tolerance, and survival.

**Table 2:** Nutrition counseling content

Section	Content
Nutrition assessment	Detailed review of weight history, food intake, physical function, nutrition-impact symptoms, micronutrient deficiencies, medical history related to nutrition, treatment plan, relevant labs
Social needs	Screening for food insecurity and other barriers to nutrition
Food preferences	Discussion about foods individual typically eats, cultural, religious, or other dietary preferences
Physical activity	Review of physical activity
Goal setting and values clarification	Determine patient goals for improving nutrition and/or function and motivation for behavior change (e.g., quality of life)
Energy and protein requirements	Estimate requirements for calorie and protein intake
Nutrition counseling and education	Provide patient with dietary strategies based on nutrition problems (e.g., meal planning with nausea), recommended interventions (e.g., supplements, referrals), and education as needed (e.g., high quality protein sources)
Follow up	Review next steps, remind patient of what should be done between visits (e.g., food and symptom diary) and timing of next appointment

**Table 3:** Pre-planned benchmarks for pilot trial

Measure	Benchmark
**Recruitment**: Proportion of eligible patients who consent to participate	≥ 50%
**Data Completion**: Proportion of patients who complete all study assessments	≥ 70%
**Retention**: Proportion of patients who are retained at the end of the intervention	≥ 85%
**Counseling adherence**: Proportion of patients who attend the majority (3/4) dietitian visits	≥ 70%
**Logging adherence**: Proportion of patients who log food intake and symptoms ≥ 3 days a week for study period	≥ 70%
**Question prompt list usage**: Proportion of patients who report using question prompt list	≥ 70%
**Educational brochure usage**: Proportion of patients who report using educational brochure	≥ 70%
**Fidelity**: Dietitian adherence to prescribed elements of counseling outlined in checklist	≥ 80%
**Acceptability**: Proportion of patients who report high satisfaction with intervention	≥ 80%
**Perceived usefulness**: Proportion of patients who report question prompt list was useful for patient-dietitian discussions	≥ 80%

## Data Availability

Data sharing is not applicable to this article as no datasets were generated or analyzed during the creation of the study protocol. Data from the clinical trial proposed in this study protocol will be made available through the UNC Dataverse data repository in the future.

## Abbreviations

CI: Confidence interval
CT: Computer tomography
DoD: Department of Defense
EHR: Electronic health record
FRAME: Framework for Reporting Adaptations and Modifications to Evidence-based Interventions
GEE: Generalized estimating equation
GI: Gastrointestinal
HIPAA: Health insurance portability and accountability act
HR: Hazards ratio
IRB: Institutional Review Board
MCID: Minimal clinically important difference
NCI: National Cancer Institute
NIH: National Institutes of Health
PI: Principal investigator
PG-SGA: Patient-generated subjective global assessment
REDCap: Research Electronic Data Capture
STRONG: Support Through Remote Observation and Nutrition Guidance
UNC: University of North Carolina
TDF: Theoretical Domains Framework
